# Optimal sequencing budget allocation for trajectory reconstruction of single cells

**DOI:** 10.1093/bioinformatics/btae258

**Published:** 2024-06-28

**Authors:** Noa Moriel, Edvin Memet, Mor Nitzan

**Affiliations:** School of Computer Science and Engineering, The Hebrew University of Jerusalem, Jerusalem 9190401, Israel; Department of Physics, Harvard University, Cambridge, MA 02138, United States; School of Computer Science and Engineering, The Hebrew University of Jerusalem, Jerusalem 9190401, Israel; Racah Institute of Physics, The Hebrew University of Jerusalem, Jerusalem 9190401, Israel; Faculty of Medicine, The Hebrew University of Jerusalem, Jerusalem 9112102, Israel

## Abstract

**Background:**

Charting cellular trajectories over gene expression is key to understanding dynamic cellular processes and their underlying mechanisms. While advances in single-cell RNA-sequencing technologies and computational methods have pushed forward the recovery of such trajectories, trajectory inference remains a challenge due to the noisy, sparse, and high-dimensional nature of single-cell data. This challenge can be alleviated by increasing either the number of cells sampled along the trajectory (breadth) or the sequencing depth, i.e. the number of reads captured per cell (depth). Generally, these two factors are coupled due to an inherent breadth-depth tradeoff that arises when the sequencing budget is constrained due to financial or technical limitations.

**Results:**

Here we study the optimal allocation of a fixed sequencing budget to optimize the recovery of trajectory attributes. Empirical results reveal that reconstruction accuracy of internal cell structure in expression space scales with the logarithm of either the breadth or depth of sequencing. We additionally observe a power law relationship between the optimal number of sampled cells and the corresponding sequencing budget. For linear trajectories, non-monotonicity in trajectory reconstruction across the breadth-depth tradeoff can impact downstream inference, such as expression pattern analysis along the trajectory. We demonstrate these results for five single-cell RNA-sequencing datasets encompassing differentiation of embryonic stem cells, pancreatic beta cells, hepatoblast and multipotent hematopoietic cells, as well as induced reprogramming of embryonic fibroblasts into neurons. By addressing the challenges of single-cell data, our study offers insights into maximizing the efficiency of cellular trajectory analysis through strategic allocation of sequencing resources.

## 1 Introduction

Single-cell RNA-sequencing (scRNA-seq) technologies, measuring the gene expression levels of cellular populations at single-cell resolution, have been instrumental in uncovering principles of development, tissue homeostasis, reprogramming, and cascades of cell decisions and their underlying mechanisms at resolutions and scales that have been inaccessible until recently (Kolodziejczyk *et al.* 2015, Hedlund and Deng 2018, Hwang *et al.* 2018, Papalexi and Satija 2018). Such dynamic analysis often requires computational methods for trajectory inference, or, the reconstruction of temporal trajectories of cellular states out of static population snapshots provided by scRNA-seq data [see recent reviews (Cannoodt *et al.* 2016, Hwang *et al.* 2018, Kester and van Oudenaarden 2018, Ding *et al.* 2022) and benchmarking analysis (Saelens *et al.* 2019)]. In practice, however, trajectory inference can be challenging; scRNA-seq data is noisy and sparse due to both stochastic biological processes and technical noise arising from experimental limitations, including constraints on the fraction of cells and reads that can be captured (Lähnemann *et al.* 2020). Moreover, extrinsic biological variation is introduced by factors that are partially independent of the temporal process, such as the physical positioning of cells (Wagner *et al.* 2016).

**Figure 1.**
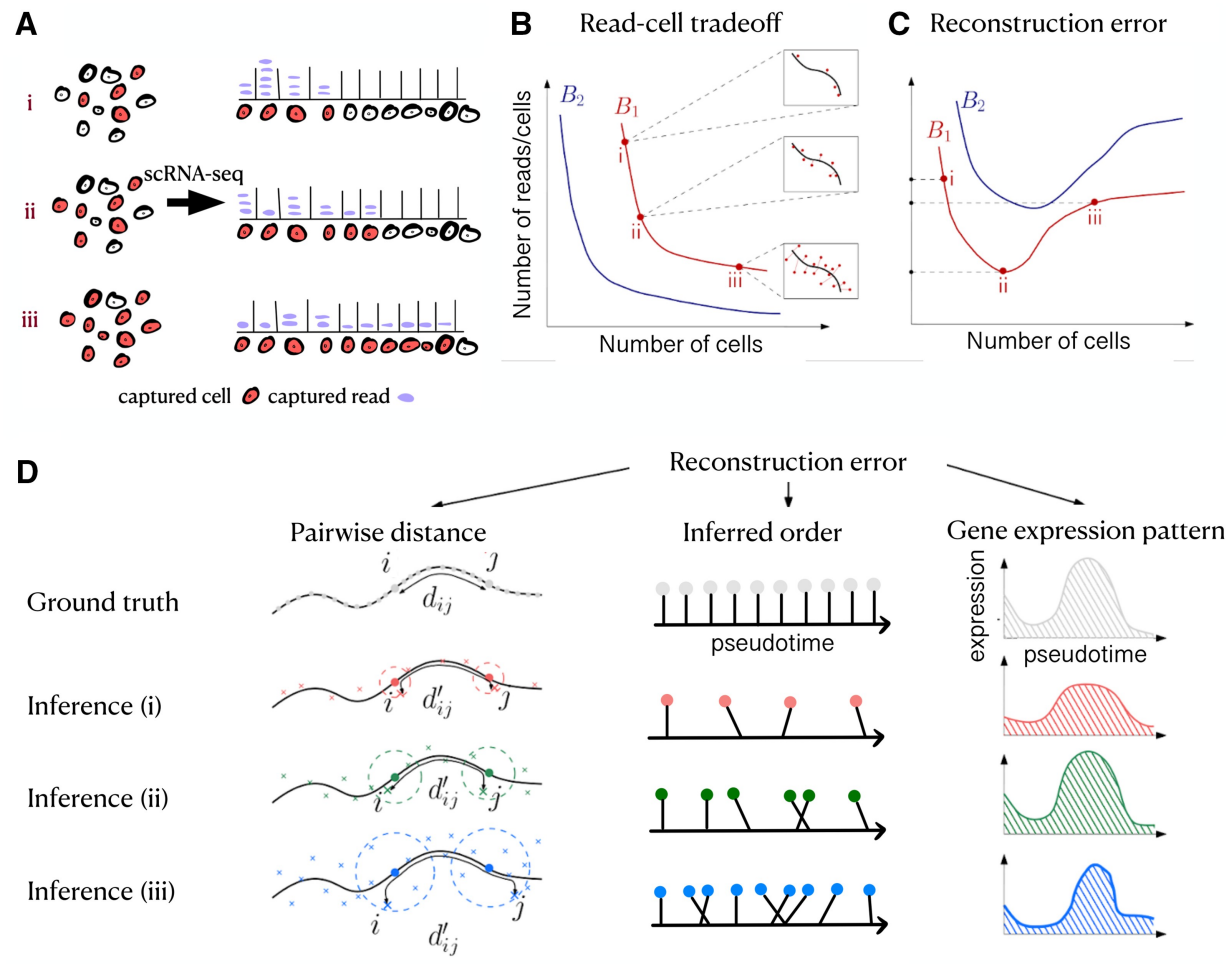
The breadth-depth tradeoff of trajectory reconstruction. (A) Under a fixed scRNA-seq sequencing budget *B*, each of the $n_{c}$ cells that are captured (red) has an average coverage of $n_{r}=B/n_{c}$ reads. An experimenter can assay (i) few cells, many reads per cell, (iii) many cells, few reads per cell, or (ii) an intermediate option. (B) Fixed sequencing budget curves, for high ($B_{1}$) and low ($B_{2}$) budgets, show the tradeoff between the number of cells and reads per cell. *(Insets)* Illustrations of cell positions (red dots) and ground truth trajectory in gene expression space (black line). (C) For a given sequencing budget, the trajectory reconstruction error can be non-monotonic with an optimum at an intermediate number of sampled cells. (D) The reconstruction error is computed *a priori* in terms of pairwise distances between cells and manifested *a posteriori* through properties of the reconstructed trajectory such as its pseudotime ordering and the patterns of gene expression along it.

Biological and technical noise can be reduced by increased biological replicates as well as improved experimental protocols and design choices (Grün and van Oudenaarden 2015, Kolodziejczyk *et al.* 2015, Stegle *et al.* 2015, Bacher and Kendziorski 2016, Ecker *et al.* 2017, Haque *et al.* 2017, Tung *et al.* 2017, Birnbaum 2018, Bass *et al.* 2019, Dal Molin and Di Camillo 2019, Seirup *et al.* 2020). In particular, technical variation can be controlled by adjusting the sequencing depth (i.e. the number of reads sequenced) (Pollen *et al.* 2014, Shalek *et al.* 2014, Streets and Huang 2014, Rizzetto *et al.* 2017, Tung *et al.* 2017). Yet, sequencing depth can only be set within experimental and financial limitations, many times at the expense of the number of cells assayed (breadth), under a given sequencing budget. This *breadth-depth tradeoff* is a fundamental experimental design challenge (Heimberg *et al.* 2016) which can be modeled in terms of a constant sequencing budget constraint, $B=n_{c}n_{r}$, where *B* is the total number of reads sequenced across all assayed cells, $n_{c}$ is the number of cells assayed, and $n_{r}$ is the average number of reads sequenced per cell (Fig. 1A and B). Thus, given a limited sequencing budget, the experimenter can navigate between acquiring noisy information (few reads per cell) for many cells and acquiring higher-quality information (many reads per cell) for fewer cells. Several recent works approached the challenge of optimizing the breadth-depth tradeoff under a fixed sequencing budget for tasks including identification of transcriptional programs (Heimberg *et al.* 2016), modeling gene expression distributions (Svensson *et al.* 2019), profiling rare cell types (Torre *et al.* 2018), and gene expression estimation (Zhang *et al.* 2020). However, optimizing this tradeoff for dynamic processes is an unresolved challenge (Ding *et al.* 2022).

Here, we approach this task by analyzing the breadth-depth tradeoff in the context of cellular trajectory reconstruction (Fig. 1C and D). We find that the accuracy of reconstruction of internal cell structure, or cell-to-cell distances in expression space, scales with the logarithm of either the sequencing depth or breadth, we show how these relate to the optimal breadth-depth allocation for a fixed sequencing budget, and we demonstrate how such choices affect trajectory reconstruction for diverse scRNA-seq datasets which capture cellular trajectories along differentiation and reprogramming. The code is available at https://github.com/nitzanlab/trajectory_reconstruction_tradeoff.

## 2 Materials and methods

### 2.1 Reconstruction error

We measure the reconstruction error as the discrepancy between the normalized pairwise geodesic distances of the complete data and the subsampled data. To compute the pairwise geodesic distances, given the raw read count matrix $X\in N^{g\;\times \;n_{c}}$, where *g* is the number of genes and $n_{c}$ is the number of cells, we first apply standard preprocessing involving logarithmic transformation using log(X + 1), followed by Principal Component Analysis (PCA) for dimensionality reduction to the top 10 principal components. This process serves as an approximation of cellular positioning in gene expression space. Subsequently, this reduced latent space representation is used to construct a *k*-Nearest Neighbors graph, wherein the value of *k* is determined as the minimum required to achieve a fully connected graph. The inferred distances between pairs of cells are computed as the shortest path distances across this graph. The reconstruction error is then calculated using the L1 norm of these normalized distances. Normalization is done by dividing each distance ($d_{ij}$ between cells *i* and *j*) by the maximal distance observed across all cell pairs (k,l), $\max_{k,l\in \mathrm{cells}}(d_{kl})$.

### 2.2 Modeling the empirical reconstruction error

The read reconstruction error, $\varepsilon_{t}$, is modeled as follows:

(2) $$\begin{array}{c} \varepsilon_{t}\approx \{\begin{array}{cc} a\;+\;b\;\text{log}\;p_{t} & \text{for}\;p_{t}\;<\;p_{t}^{\mathrm{sat}}\\ \varepsilon_{t}^{\mathrm{sat}} & \text{otherwise} \end{array}. \end{array}$$

where $p_{t}^{\mathrm{sat}}$ denotes the transition into a saturated sequencing regime where the reconstruction error, $\varepsilon_{t}^{\mathrm{sat}}$, is approximately constant.

The cell reconstruction error, $\varepsilon_{c}$, is fitted to the fraction of captured cells, $p_{c}$, by $\varepsilon_{c}\approx \alpha \;+\;\beta \;\text{log}\;p_{c}$.

In both cases, a,b,α, and β are constants that parameterize the relationships between the subsampling rates and the respective reconstruction errors.

Based on empirical results (see Supplementary Fig. S7), we model the overall reconstruction error as $\varepsilon =\max(\varepsilon_{c},\varepsilon_{t})$. Consequently, to predict the optimal cell subsampling probability, $\hat{p_{c}}$, we solve $\varepsilon_{c}=\varepsilon_{t}$ for $p_{c}$. Given $\tilde{B}$, the fractional sequencing budget, and $p_{c}$, the cell subsampling probability, the respective cell reconstruction error is $\varepsilon_{c}=\alpha \;+\;\beta \;\text{log}\;p_{c}$. When sequencing is unsaturated ($p_{t}\;<\;p_{t}^{\mathrm{sat}}$), the estimated read reconstruction error is $\varepsilon_{t}=a\;+\;b\;\text{log}\;p_{t}=a\;+\;b\;\text{log}\;\frac{\tilde{B}}{p_{c}}$, and $\hat{p_{c}}=\text{exp}(\frac{a\;-\;\alpha \;+\;b\;\text{log}\;\tilde{B}}{\beta \;+\;b})$. When sequencing is saturated ($p_{t}\;\geq \;p_{t}^{\mathrm{sat}}$), sequencing at read subsampling probability $p_{t}^{\mathrm{sat}}$ is optimal. Hence, altogether:

(3) $$\begin{array}{c} \hat{p_{c}}∼\{\begin{array}{cc} {\tilde{B}}^{\gamma } & p_{t}\;<\;p_{t}^{\mathrm{sat}}\\ \tilde{B}/p_{t}^{\mathrm{sat}} & \text{otherwise} \end{array}, \end{array}$$

with $\gamma =\frac{b}{\beta \;+\;b}$.

In Fig. 3B, the inferred optimal cell subsampling probability, $\hat{p_{c}}$, is contrasted with the empirical optimal cell subsampling probability, $p_{c}^{*}$. To get the empirical optimum $p_{c}^{*}$ per budget $\tilde{B}$, we compute the reconstruction error for $p_{c}\in [0.01,0.9]$ (and corresponding $p_{t}=\frac{\tilde{B}}{p_{c}}$), thresholding at a minimum of 5 cells and 20 reads on average per cell, see Fig. 3A for reconstruction error tradeoff curves. The empirical optimum $p_{c}^{*}$ is the cell subsampling probability corresponding to the minimal average reconstruction error, averaged over 50 repetitions and over a rolling window of 4 $p_{c}$ values.

### 2.3 Pseudotime labeling of linear trajectories with diffusion pseudotime

We follow a basic pipeline suggested in Scanpy (Alexander Wolf *et al.* 2018) of ordering cells by diffusion pseudotime based on (Haghverdi *et al.* 2016). This involves preprocessing the data (Zheng *et al.* 2017), computing the neighborhood graph over reduced principal component representation, while avoiding disconnected components by increasing the number of neighbors when such exist, and computing the corresponding diffusion map and the resulting pseudotime ordering of the cells. Alternative pseudotime ordering methods are described in the Supplementary data.

### 2.4 Computing ordered expression pattern over linear trajectories

For this analysis, we focus on the 50 genes exhibiting the highest expression levels (averaged over all cells) from the top 10% highly variable genes chosen with Scanpy (Alexander Wolf *et al.* 2018) (Section 2). For each gene, we compute its ordered expression pattern by first binning cells into 5 equally sized groups according to their pseudotime ordering of the full data (see Section 2 and Supplementary Notes). Then, each gene’s expression is averaged per bin and normalized by the bin with maximal expression. In-silico runs in which no reads of the gene are captured are ignored. For each gene, we compute the Pearson correlation of the normalized, ordered expression of the subsampled and full data and report for each subsampling experiment the averaged correlation across genes.

### 2.5 Inferring gene expression patterns over linear trajectories

We follow the same procedure as for computing the ordered expression pattern (see Section 2), except that instead of binning cells by their pseudotime ordering of the full data (considered as ground-truth ordering), we use the inferred pseudotime ordering of the subsampled data, using pseudotime inference methods as described in the Section 2 and in the Supplementary data.

## 3 Results

### 3.1 Reconstruction error under subsampling of cells or reads per cell

The quality of the reconstruction of a dynamical process can depend on the quality of the scRNA-seq data, the characteristics of the underlying trajectory, and the specific trajectory inference algorithm used (Kester and van Oudenaarden 2018, Saelens *et al.* 2019, Wagner and Klein 2020). Since many trajectory inference methods, such as PAGA (Alexander Wolf *et al.* 2019) and Wanderlust (Bendall *et al.* 2014), are based on a computation of pairwise distances (Kester and van Oudenaarden 2018) over a low dimensional representation of cells [e.g. using log transformation and PCA, as advocated in (Ahlmann-Eltze and Huber 2023)], we center our analysis around the estimation quality of pairwise geodesic distances of such cell embeddings. Specifically, we examine the impact of subsampling cells or reads per cell on inferred pairwise distances $d_{ij}^{\;{\;}^{\prime }}$ between cells *i*, *j*, computed as shortest path distances over k-Nearest Neighbor graph of the cells, relative to the distances $d_{ij}$ before subsampling, which we take as a proxy for the ground truth (see Section 2). That is, we quantify the *reconstruction error* ε in terms of the discrepancy between the inferred and ground-truth cell-to-cell distances:

(1) $$\varepsilon =\frac{1}{n_{c}^{2}}\sum_{ij}\left|{\hat{d}}_{ij}-{\hat{d}}_{ij}^{\;{\;}^{\prime }}\right|,$$

where ${\hat{d}}_{ij}$ and ${\hat{d}}_{ij}^{\;{\;}^{\prime }}$ are both scaled entry-wise to a maximum of 1 (${\hat{d}}_{ij}=d_{ij}/{\text{max}}_{\text{kl}}(d_{kl})$).

We begin by studying how subsampling only cells or only reads per cell (while the other quantity remains fixed) affects the quality of trajectory reconstruction. For either read or cell subsampling (binomial sampling of reads and uniform random sampling of cells; see examples in Supplementary Fig. S5), we vary either $p_{t}$, the fraction of read counts ($p_{t}=n_{r}/n_{r}^{0}$), or $p_{c}$, the fraction of cells captured ($p_{c}=n_{c}/n_{c}^{0}$), and compute the reconstruction error of the pairwise geodesic distances between cells in the subsampled data, relative to their ground-truth distances. We denote by $\varepsilon_{t}$ (or $\varepsilon_{c}$) the reconstruction error when subsampling only reads (or only cells). Of note, when a Unique Molecule Identifier (UMI) is incorporated in the RNA-sequencing protocol, multiple reads can be understood as originating from a single transcript (leading to differences in the subsampling of reads versus transcripts, particularly for deep sequencing). Here, we use reads and transcripts interchangeably to represent the captured expression as done in (Zhang *et al.* 2020).

**Figure 2.**
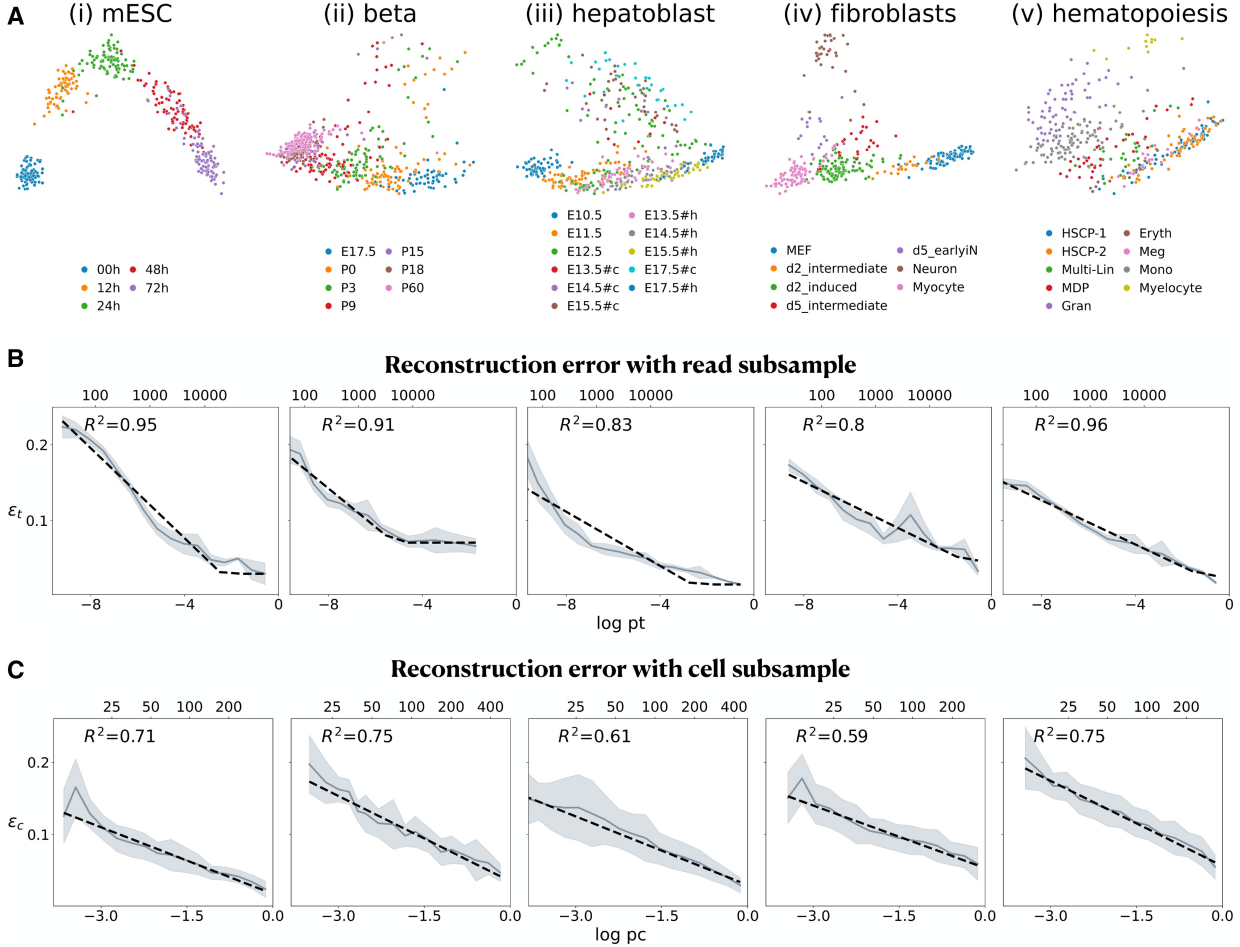
The independent effects of subsampling reads/cells on the accuracy of inferred cell-cell distances. (A) scRNA-seq datasets used for evaluation, embedded and plotted in the first two principal components following log transform (see Section 2) and colored by “milestones” as standardized in Saelens *et al.* (2019). (B) The resulting reconstruction error $\varepsilon_{t}$ decreases linearly with $\text{log}\;p_{t}$ (fitted by a dotted black line), where $p_{t}$ is the fraction of sampled read counts. Large $p_{t}$ denote a regime of saturated sequencing in which the error $\varepsilon_{t}^{\mathrm{sat}}$ is modeled as constant. Mean number of reads per cell ($n_{r}$) are noted on the top *x*-axis. (C) The reconstruction error $\varepsilon_{c}$ decreases linearly with $\text{log}\;p_{c}$ (fitted by a black dotted line) where $p_{c}$ is the fraction of sampled cells. Number of cells ($n_{c}$) are noted on the top *x*-axis. In both (B) and (C), subsampling is performed 10 times, mean error is denoted by a gray line, standard deviation is denoted by a shaded region. $R^{2}$ (coefficient of determination) is computed between each sample and the fitted curve. Titles in B and C correspond to the titles of the columns in (A).

We analyzed five publicly available, deeply sequenced, mouse-derived single-cell datasets, including those of embryonic stem cells differentiation into primitive endoderm cells [“mESC”; (Hayashi *et al.* 2018)], maturation of pancreatic beta cells [“beta”; (Qiu *et al.* 2017)], hepatoblasts differentiation into hepatocytes and its branched cholangiocytes [“hepatoblast”; (Yang *et al.* 2017)], embryonic fibroblasts reprogramming into induced neuronal or myocyte cells [“fibroblasts”; (Treutlein *et al.* 2016)], and transitioning of multipotent hematopoietic cells towards lineage-specific progenitors [“hematopoiesis”; (Olsson *et al.* 2016)], see Fig. 2A and Supplementary Table S1 for further details.

Across these datasets, the reconstruction error ε decreases approximately linearly with $\log(p_{c})$ (Fig. 2C; mean $R^{2}=0.68$), as well as with $\log(p_{t})$ (Fig. 2B; mean $R^{2}=0.89$) for $p_{t}\;<\;p_{t}^{\mathrm{sat}}$, beyond which is a regime dominated by sequencing saturation, where $\varepsilon (p_{t}\;\geq \;p_{t}^{\mathrm{sat}})=$ constant (see Section 2 for details). While we focus on deeply sequenced datasets (Fig. 2), which are respectively associated with small sample sizes (355–562 cells per dataset), the reconstruction error scales similarly with cell subsampling for larger datasets, as we show for scRNA-seq data of pancreatic endocrinogenesis composed of 3696 cells (Bastidas-Ponce *et al.* 2019) (Supplementary Fig. S6).

### 3.2 Reconstruction error under sequencing budget constraints

Given a fixed sequencing budget, *B*, how can one minimize the reconstruction error? Concretely, what is the optimal number of cells to assay, $n_{c}^{*}$, alternatively modeled as the optimal cell subsampling probability, $p_{c}^{*}$, that achieves minimal reconstruction error? The inherent tradeoff between the number of cells and the number of reads per cell given a constant budget *B* (where $B=n_{c}n_{r}$) suggests that any shift in the balance between sequencing breadth and depth would lead to conflicting effects on the reconstruction error, as individually, the error increases with subsampling of both cells and reads (Fig. 2B and C).

**Figure 3.**
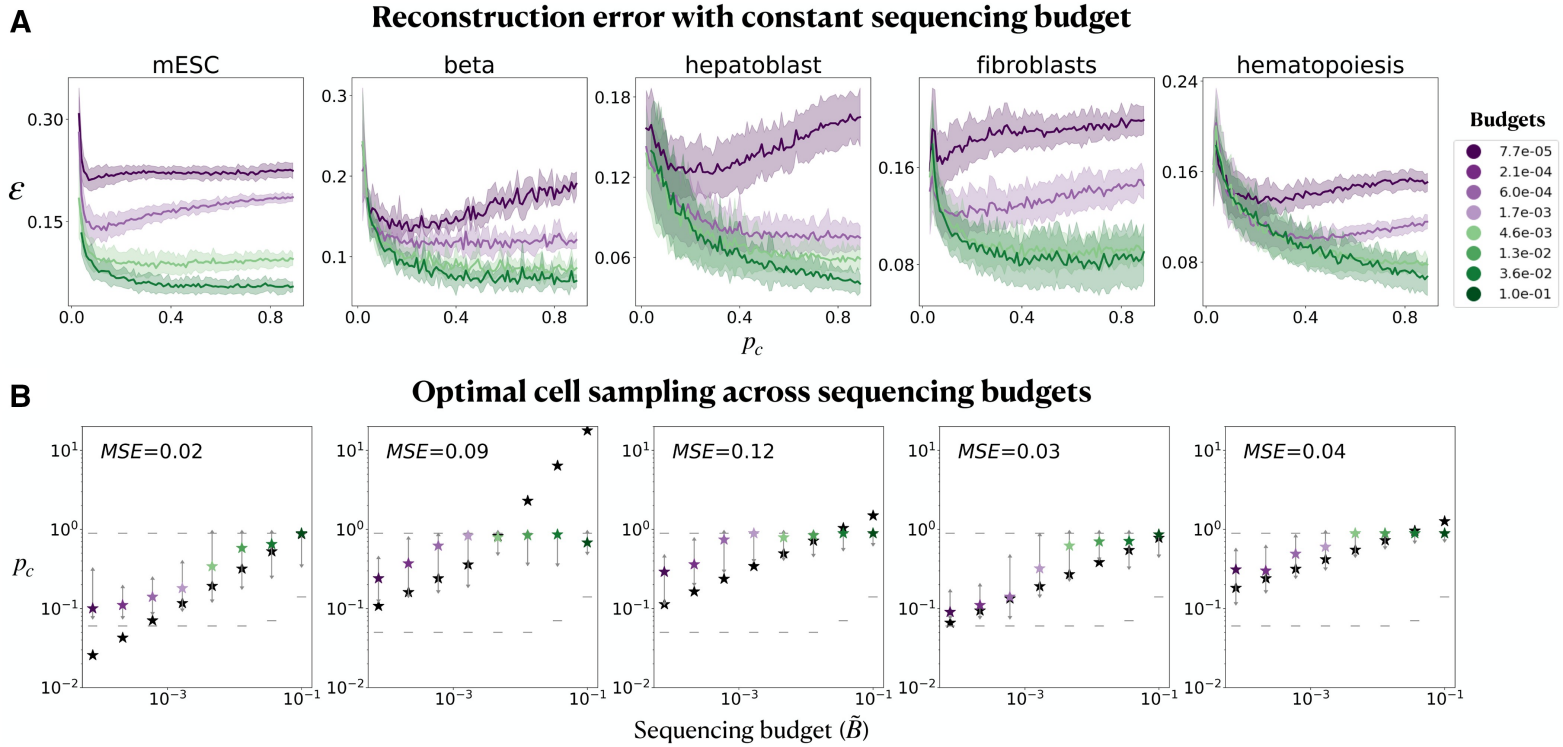
Reconstruction error and optimal sampling across fixed sequencing budgets. (A) Reconstruction error as a function of sampled cell fraction, where each curve corresponds to a different sequencing budget. Solid lines represent the mean error and the shaded regions represent standard deviation over 50 repetitions. (B) For an extended series of sequencing budgets, we measure the empirical optimal cell sampling probability, $p_{c}^{*}$ (colored stars, see budget legend in A), mark the range of $p_{c}$ whose reconstruction error is 0.01 away from optimal (gray arrows), and the minimal and maximal tested $p_{c}$ in dashed gray lines. Sequencing budgets are written in their fractional form, $\tilde{B}=B/B^{0}$ (*x*-axis and colors). From Equation 3 (see Section 2), we compute the predicted optimal cell sampling probability, $\hat{p_{c}}$ (black stars). Mean Squared Error (MSE) is computed over predicted ($\hat{p_{c}}$) and empirical ($p_{c}^{*}$) optimal cell sampling probabilities where prediction is within the range of $p_{c}$. Titles in B correspond to the titles of the columns in (A).

To study this tradeoff, we examine the reconstruction error when subsampling cells and reads for the scRNA-seq datasets described above under constant budgets (Fig. 3A). That is, we subsample a fraction of the total number of cells $n_{c}^{0}$ and of the total number of reads $n_{r}^{0}$ in the original data in order to synthetically simulate less favorable experimental conditions of a limiting sequencing budget *B* that is strictly smaller than $B^{0}=n_{c}^{0}n_{r}^{0}$. We then compute the reconstruction error over a range of cell sampling probabilities $p_{c}$ (in other words, over a range of cell numbers and corresponding read numbers).

At low sequencing budgets, the reconstruction error shows non-monotonicity as a function of $p_{c}$, and is minimized at small sampled cell fractions (Fig. 3A). For example, for the mouse embryonic stem cell data (Hayashi *et al.* 2018), with a budget of B≈60K reads (or equivalently, fractional sequencing budget of $\tilde{B}=B/B^{0}=6.0e\;-\;4$), the reconstruction error is minimized when sampling $n_{c}^{*}\approx 60$ cells with $n_{r}^{*}\approx 1000$ reads per cell. As the budget increases, the optimal number of cells to assay increases (Fig. 3A).

To model how the optimal subsample of cells, $p_{c}^{*}$, that minimizes the reconstruction error ε, varies with the sequencing budget *B*, we approximate ε by the maximum over both cell and read errors ($\varepsilon =\max(\varepsilon_{t},\varepsilon_{c})$), supported empirically in Supplementary Fig. S7. Using this modeling assumption, the reconstruction error is expected to be minimal when $\varepsilon_{t}=\varepsilon_{c}$. Hence, when sequencing is unsaturated, the predicted optimal number of cells to assay, $\hat{p_{c}}$, follows a power-law, $\hat{p_{c}}∼{\tilde{B}}^{\gamma }$ where $\tilde{B}$ is the fractional sequencing budget, $\gamma =\frac{b}{\beta \;+\;b}$, and b,β are inferred based on the fit of reconstruction error as a function of $p_{c}$ and $p_{t}$, individually (Section 2). These predictions for optimal allocation of sequencing budgets ($\hat{p_{c}}$) are found to be in good agreement with the empirical optimal allocations ($p_{c}^{*}$) for the five scRNA-seq datasets presented above (Fig. 3B; mean MSE=0.06).

### 3.3 Breadth-depth sequencing tradeoff is reflected in trajectory reconstruction for cells and gene expression

**Figure 4.**
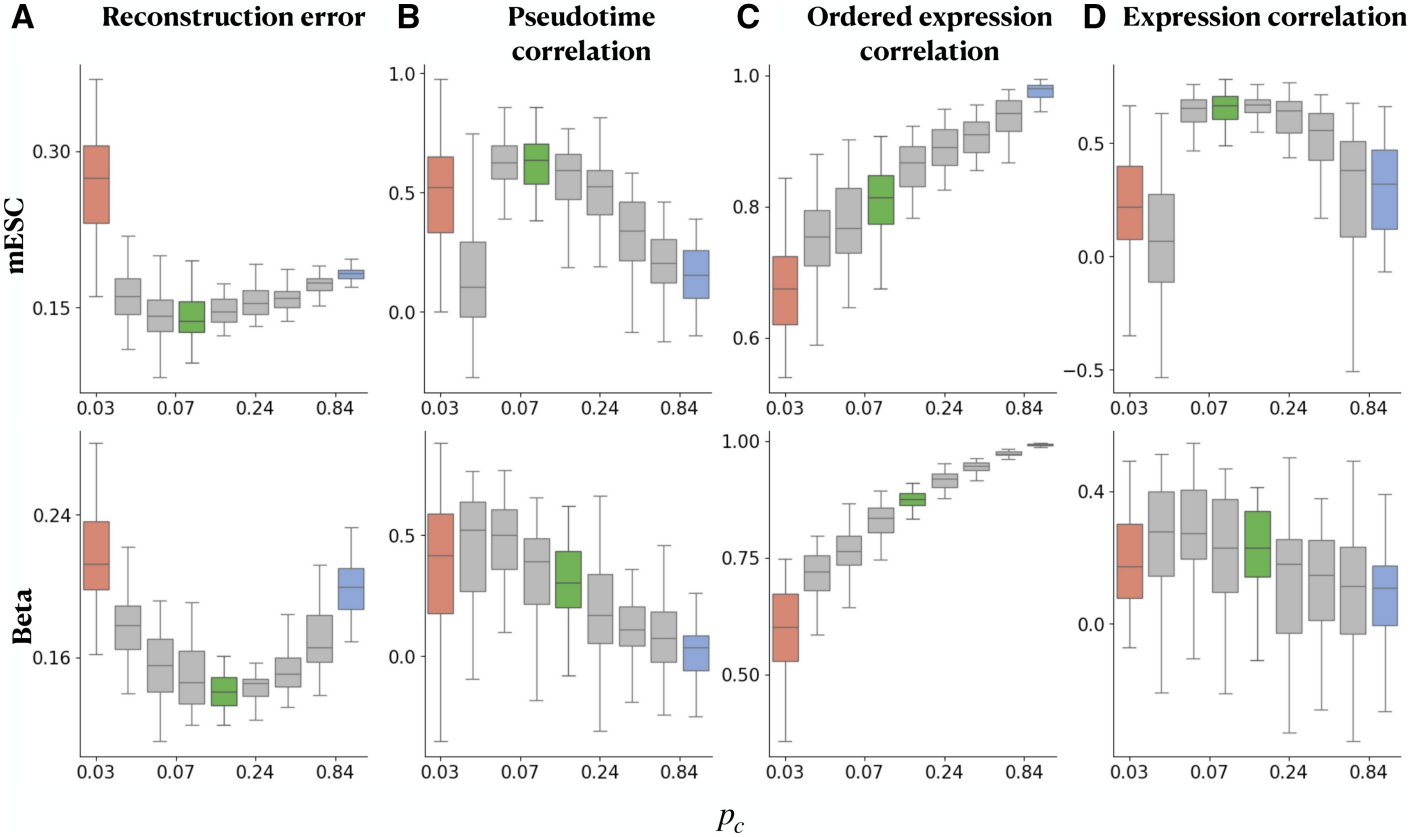
Low reconstruction error for intermediate cell-read budget allocation manifests in downstream tasks. We analyze the mESC and beta cells datasets using fractional budgets $\tilde{B}=6.0e-04,\tilde{B}=7.7e-05$, respectively. We compute (A) the reconstruction error, together with the Pearson correlation between the complete and subsampled data in terms of: (B) diffusion pseudotime, (C) gene expression patterns, given the true pseudotime (ordering of the full data), (D) gene expression patterns over the inferred pseudotime (see Section 1). Throughout, we highlight sampling experiments of minimal reconstruction error (green) and of deeper or broader choices marked in red or in blue, respectively.

To assess the downstream effects of the breadth-depth sequencing tradeoff on trajectory reconstruction beyond cell-cell distances, we next analyzed the inferred gene expression pattern of highly expressed and variable genes across linear differentiation trajectories, see Fig. 4. Inferring the change in gene expression across a reconstructed linear trajectory requires (i) accurate pseudotime ordering of the cells (Fig. 4B), and (ii) high-quality capture of the expression pattern (Fig. 4C). When both are achieved, the quality of the inferred expression pattern across the reconstructed trajectory is expected to be high (Fig. 4D). See Section 2 for further details.

We find that using the sequencing budget to sequence many cells at low read counts (high breadth, low depth) can impede the recovery of the arrangement of cells for both mouse ESC and beta cell differentiation datasets (Fig. 4B). On the other hand, given the cellular ordering along the trajectory, estimation of the corresponding mean gene expression can benefit from a large batch of samples (Fig. 4C, see Supplementary data). While each of these substeps, individually, may benefit from lending the sequencing budget towards either deeper, or broader sampling, the complete task of recovering gene expression pattern benefits from an intermediate budget allocation (Fig. 4D). This result captures the tradeoff within the temporal gene expression inference task, demonstrated in Fig. 4B, C, as inference of cellular ordering is generally disrupted when capturing many cells and few reads per cell, and gene expression inference is disrupted when deeply sampling few cells. We obtain similar results using alternative pseudotime inference methods, see Supplementary Figures S8 and S9.

## 4 Discussion

In this paper, we analyzed the optimal allocation of a sequencing budget in single-cell RNA-sequencing experiments so as to optimize cellular trajectory reconstruction. Such optimization can play a key role both for smaller-scale pilot experiments which serve to inform the design of subsequent larger-scale studies, or high-throughput screenings where efficient use of resources is key. While the optimal tradeoff can depend on biological features such as the precise topology of the underlying trajectory, or the rates at which cells progress along the trajectory, we abstract away much of this intricacy by modeling directly the change of reconstruction error with read or cell subsampling. The combined effect of subsampling cells and reads can be complex, however, we find that the reconstruction error, to a good approximation, is structured according to the factor corresponding to the dominant error, thus providing interpretation of the emerging reconstruction error over the breadth-depth tradeoff and a prediction of the optimal experimental design.

While in this work we focus on optimizing sequencing budget allocation to enhance the quality of trajectory reconstruction, different objectives can lead to substantial variations in the optimal budget allocation strategy. For example, Heimberg *et al.* (2016) showed that shallow sequencing can suffice for the recovery of transcriptional programs due to the modularity of gene expression, and specifically the error of the inferred programs, or the low-dimensional structure of the data, saturates with increasing depth, starting at relatively low depth. When targeting genes of exceedingly low expression, Zhang *et al.* (2020) suggest to assay extremely few cells, a strategy which can be suboptimal for trajectory reconstruction (see Supplementary data).

While we demonstrate several advantages of our chosen budget with regard to the cellular trajectory, our study can be broadened to address multi-faceted objectives (e.g. preserving topology and gene expression pattern of specific genes) and to be posed as a satisfaction, rather than an optimization, problem. Our approach can be generalized to additional biological structures or processes, such as optimizing the breadth-depth tradeoff for the inference of the spatial configuration of tissues, and integrated with structural and expression-based prior knowledge.

## Supplementary Material

btae258_Supplementary_Data

## Data Availability

Single-cell datasets of mESC differentiation into primitive endoderm cells, pancreatic beta cells maturation, hepatoblasts bifurcating differentiation, embryonic fibroblasts reprogramming, and of hematopoiesis were downloaded from https://zenodo.org/record/1443566#.YEExrpMzbDI (Saelens *et al.* 2019). The pancreatic endocrinogenesis dataset (Bastidas-Ponce *et al.* 2019) was downloaded through scVelo (Bergen *et al.* 2020). See dataset statistics in Supplementary Table S1.

## Code availability

Code is available at: https://github.com/nitzanlab/trajectory_reconstruction_tradeoff.
