# Supplementary material for: Optimal sequencing budget allocation for trajectory reconstruction of single cells
Source: Bioinformatics. 2024 Jun 28;40(Suppl 1):i446–52. doi: 10.1093/bioinformatics/btae258 (PMC11211845; doi:10.1093/bioinformatics/btae258)
Supplement: btae258_Supplementary_Data [file btae258_supplementary_data.pdf]

Supplementary Material

Error of gene expression estimation

We study the performance of the optimal read-cell allocation in terms of the reconstruction error in recovering gene expression patterns along a linear trajectory. We decompose this task into two steps: 1. ordering cells, 2. estimating gene expression pattern along the trajectory given the ordering of the cells. For the latter step, given a dataset, we infer its corresponding gene expression along a trajectory by bucketing the cells according to their pseudotime ordering in the full data (considered as ground-truth ordering) and computing the mean gene expression per bucket. We do this for both, the full data and the subsampled data and compute the correlation of gene expression between the two cases, see Fig. 4C.

For a dataset subsampled with cell and transcript probabilities p_c, p_t (respectively), we examine the variance of the mean estimated per bucket as an approximation of the inferred expression error compared to the mean expression of the complete data. Let μ^0_{bg} and σ^0_{bg} be the mean and variance of expression of gene g in bucket b for the full data, and μ_{bg} and σ_{bg} be the mean and variance expression computed for the same bucket after subsampling, respectively. Let X_{cg} be the expression of gene g in cell c. Under binomial sampling of reads, the variance of X_{cg} is : $Var[X_{cg}] = p_t^2\sigma^0_{bg} + p_t(1-p_t)\mu^0_{bg}$. Assuming the expression of cells within the bucket are sampled i.i.d, the variance of the sampled mean expression is $Var[\mu_{bg}] = \frac{p_t^2\sigma^0_{bg}+p_t(1-p_t)\mu^0_{bg}}{n^0_{cb}p_c} \sim \frac{1}{B}\left(p_t^3\sigma^0_{bg} + p_t^2(1-p_t)\mu^0_{bg}\right)$, where n^0_{cb} is the number of cells in bucket b. Consequently, the derivative of $Var[\mu_{bg}]$ with respect to p_t is: $\frac{\partial Var[\mu_{bg}]}{\partial p_t} = \frac{1}{B}\left(3(\sigma^0_{bg} - \mu^0_{bg})p_t^2 + 2\mu^0_{bg}p_t\right)$.

When $\sigma^0_{bg} \geq \mu^0_{bg} > 0$, the derivative $\frac{\partial Var[\mu_{bg}]}{\partial p_t} \geq 0$ and is equal to zero only for $p_t = 0$, and so the variance of μ_{bg} increases with p_t. When $0 < \sigma^0_{bg} < \mu^0_{bg}$, the derivative has two roots: $p_t = 0$ and $p_t = \frac{2\mu^0_{bg}}{3(\mu^0_{bg}-\sigma^0_{bg})}$. $p_t = \frac{2\mu^0_{bg}}{3(\mu^0_{bg}-\sigma^0_{bg})}$ is within the range of $[0,1]$ only for $\sigma^0_{bg} < \frac{\mu^0_{bg}}{3}$ and the derivative is then negative where $p_t \in (0, \frac{2\mu^0_{bg}}{3(\mu^0_{bg}-\sigma^0_{bg})})$.

This means that the variance of μ_{bg} increases with p_t for $p_t \in [0,1]$, except when the variance is substantially smaller than the mean (less than a third of the mean) and $p_t \in (0, min(1, \frac{2\mu^0_{bg}}{3(\mu^0_{bg}-\sigma^0_{bg})}))$. Considering the variance of the mean estimation as a proxy of the error in gene expression estimation, we conclude that in most cases, the quality of the inferred expression pattern improves as we allocate the budget to assay more cells.

Pseudotime ordering with alternative trajectory inference methods

Regression We conduct a similar projection to that presented in [26]. Namely, for n cells and g genes, given $y \in \mathbb{R}^{g\times n}$ the expression of the cells and $A \in \mathbb{R}^{g\times p}$ the mean expression profiles of the start and end milestones ($p = 2$), we compute $w = y^T A(A^T A)^{-1} \in \mathbb{R}^{n\times p}$, where w represents, for each cell, its weights relative to the start and end milestones. We use the relative weight to the start milestone as the pseudo order.

Component1 We use the first PC component (after log transformation) as a proxy of pseudotime ordering, an approach proposed in [29] for linear trajectories. We take the reverse order when the embedding is flipped.

PAGA We apply a pipeline suggested in Scanpy [41] including preprocessing of the data [45], computing the neighborhood graph over reduced principal component representation, while avoiding disconnected components by increasing the number of neighbors when such exist, clustering the data using the Louvain algorithm [36], computing communities' graph abstraction with PAGA [42], followed by diffusion pseudotime computation [13].

Challenges in simultaneously recovering trajectory structures and expression of lowly expressed genes under sequencing budget tradeoff

Identifying the expression of biologically important, yet lowly expressed, genes, such as developmental transcription factors, is critical. However, the budget allocation strategy that optimizes trajectory reconstruction, proposed in this study, may be suboptimal in capturing the expression of these genes. For instance, in the mESC dataset, functionally important genes Sox2 and Gata4 are captured at 13.6 and 8.4 reads per cell, respectively. In a related work, Zhang et al. study the breadth-depth tradeoff, focusing on the accurate recovery of the expression statistics of a gene or a set of genes under a limited sequencing budget [44]. According to their findings, for a sequencing budget of 60K reads, it is ideal to sequence 2-3 cells with 20-30K reads each such that Sox2 and Gata4 are captured at rates of 1.6 and 1 read per cell, respectively. Under our proposed budget allocation for optimized trajectory reconstruction —sequencing 60 cells with an average of 1000 reads per cell— the rate of detecting Sox2 and Gata4 falls to 0.05 and 0.03 reads per cell, respectively. This discrepancy highlights a significant challenge: when gene expression levels are exceedingly low, our budgeting strategy may not align with the objective of capturing lowly expressed genes.

Table 1. scRNA-seq datasets analyzed in the current study.

	Technology	Trajectory type	Num cells	Num genes (K)	Num reads (M)	Reads per cell (K)	Ref
mESC	RamDA-seq	linear	414	23.66	101.46	245.07	[15]
beta	smart-seq2	linear	562	6.14	444.53	790.99	[27]
hepatoblast	smart-seq2	bifurcation	504	6.14	394.12	781.98	[43]
fibroblasts	fluidigm c1	bifurcation	355	3.38	47.57	133.99	[37]
hematopoiesis	fluidigm c1	linear	376	3.59	137.18	364.85	[23]
pancreas	10X chromium	bifurcation	3696	28.00	24.67	6.68	[4]

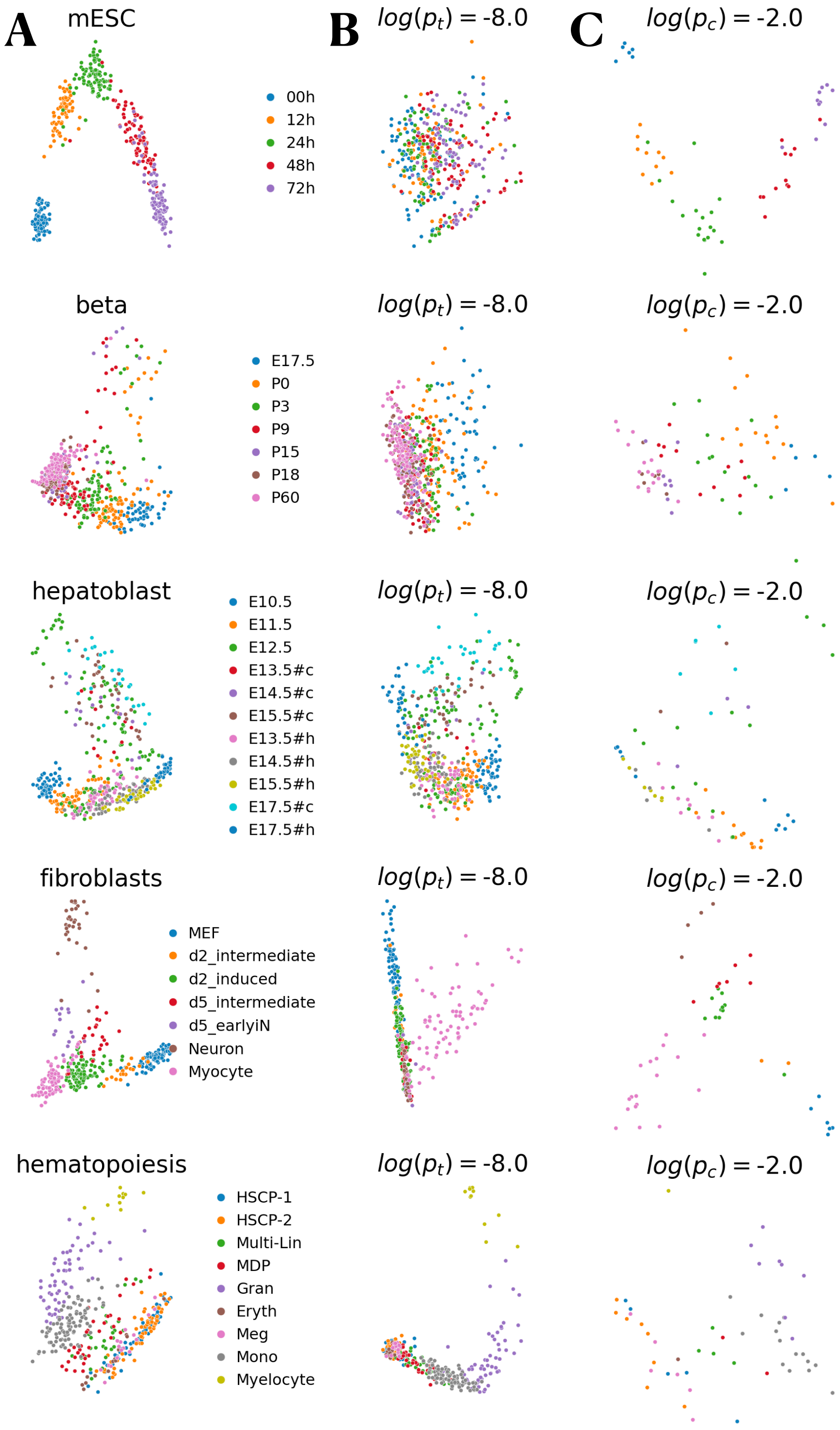

Fig. 5. Examples of subsampling experiments for each scRNA-seq dataset, where cells are embedded and plotted in the first two principal components following log transform (see Methods) and colored by their milestones [29] (A), when only reads (B) or only cells (C) are subsampled. Datasets in columns B and C correspond to those in column A.

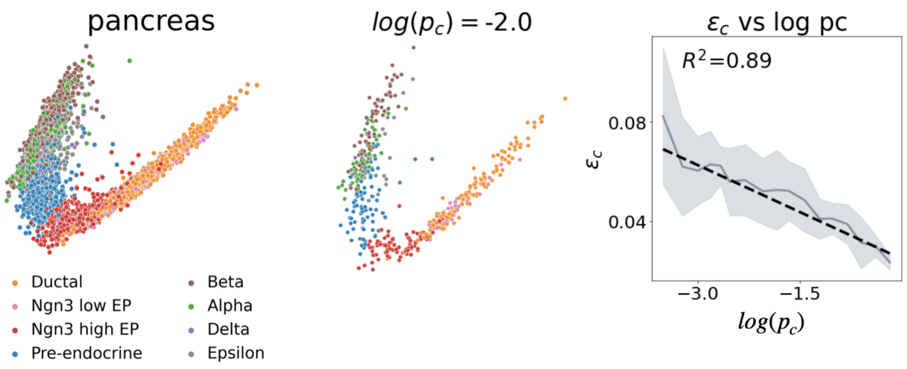

Fig. 6. Pancreatic endocrinogenesis scRNA-seq dataset from [4] which includes 3696 cells (left panel), where cells are embedded and plotted in the first two principal components following log transform (see Methods) and colored by cell type (left and middle panels). When subsampling cells at probability p_c (example shown in middle panel), reconstruction error scales linearly with $log(p_c)$ (right panel) as shown in the main text for smaller datasets (Fig. 2C). Solid line represents mean error, shaded region represents the standard deviation over 50 repetitions, dashed black line marks the linear fit, and R^2 is the coefficient of determination between the mean error and the linear fit.

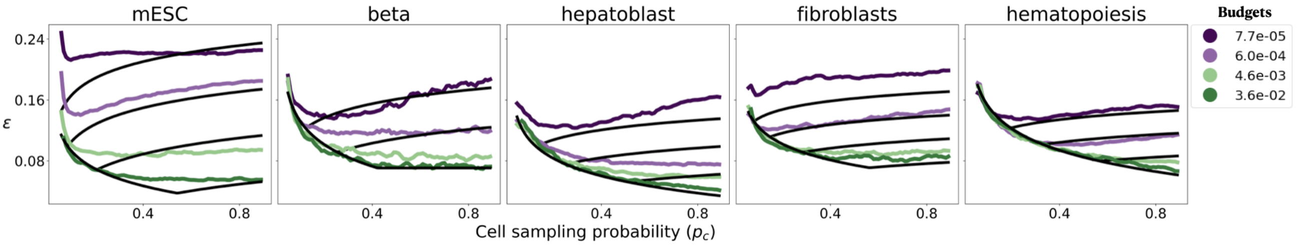

Fig. 7. Mean reconstruction error vs. cell subsampling probability for five scRNA-seq datasets, plotted for the four budgets corresponding to Fig. Fig. 3A in the main text. Reconstruction error was computed using a running average of each four consecutive points. Using the linear fits of read subsampling (Fig. 2B) and of cell subsampling (Fig. 2C) for $\varepsilon_t, \varepsilon_c$ evaluation, respectively, we plot the maximum of these two separate predicted reconstruction errors (black).

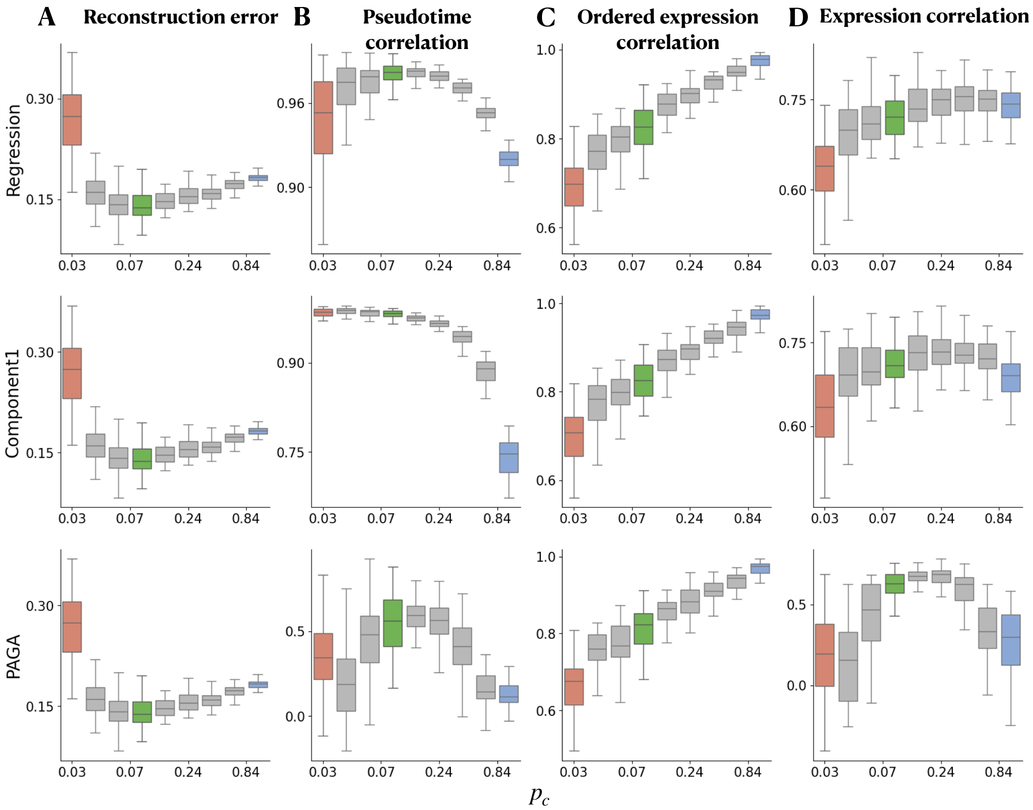

Fig. 8. Trajectory reconstruction under breadth-depth sequencing tradeoff for alternative trajectory reconstruction methods for the mESC dataset. Showing: (A) reconstruction error, (B) Pearson correlation between the pseudotime reconstruction of the full and subsampled data, (C) Pearson correlation between the gene expression patterns of the full and subsampled data given the pseudotime ordering of the full data, and (D) Pearson correlation between the gene expression patterns over the inferred pseudotime of the full and subsampled data (see Methods). Rows correspond to alternative pseudotime reconstruction methods: regression [26] (top), reconstruction based on the first principal component [29] (middle), and PAGA [42] (bottom). We highlight sampling experiments corresponding to minimal reconstruction error (green) and of deeper or broader choices marked in red or in blue, respectively.

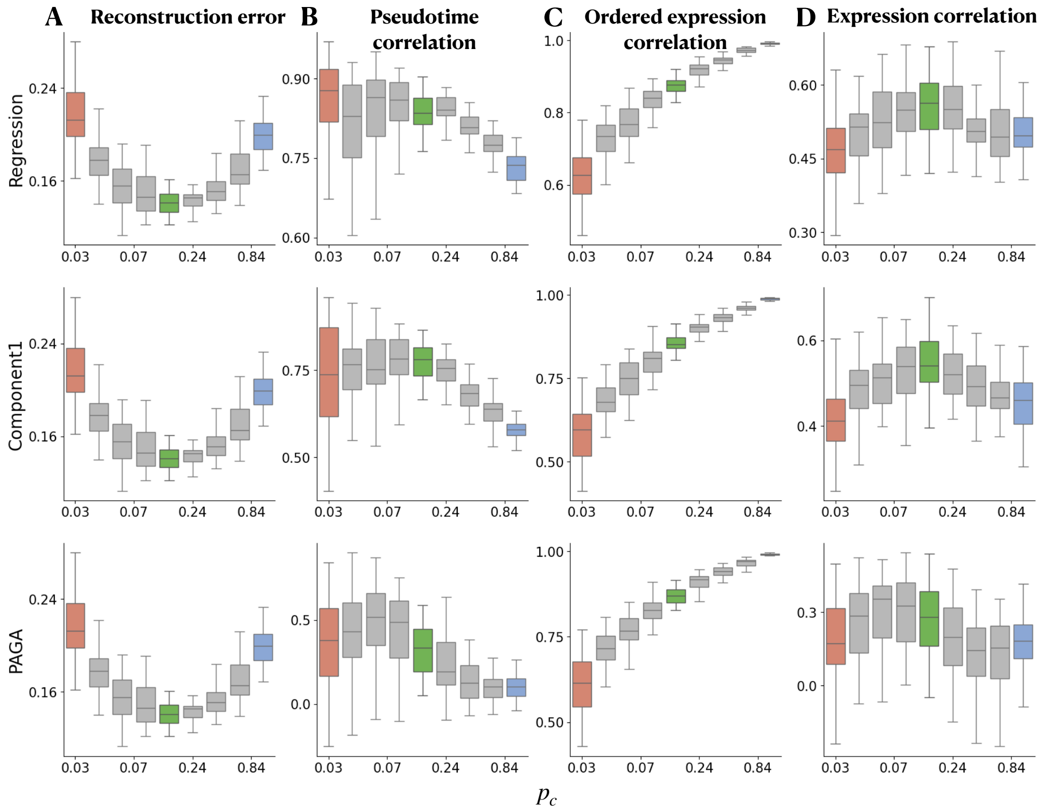

Fig. 9. Trajectory reconstruction under breadth-depth sequencing tradeoff for alternative trajectory reconstruction methods for the beta cells dataset. Showing: (A) reconstruction error, (B) Pearson correlation between the pseudotime reconstruction of the full and subsampled data, (C) Pearson correlation between the gene expression patterns of the full and subsampled data given the pseudotime ordering of the full data, and (D) Pearson correlation between the gene expression patterns over the inferred pseudotime of the full and subsampled data (see Methods). Rows correspond to alternative pseudotime reconstruction methods: regression [26] (top), reconstruction based on the first principal component [29] (middle), and PAGA [42] (bottom). We highlight sampling experiments corresponding to minimal reconstruction error (green) and of deeper or broader choices marked in red or in blue, respectively.
